# The future of PrEP among transgender women: the critical role of gender affirmation in research and clinical practices

**DOI:** 10.7448/IAS.19.7.21105

**Published:** 2016-10-18

**Authors:** Jae M Sevelius, Madeline B Deutsch, Robert Grant

**Affiliations:** 1 Center of Excellence for Transgender Health, University of California, San Francisco, San Francisco, CA, USA; 2 Center for AIDS Prevention Studies, Department of Medicine, University of California, San Francisco, San Francisco, CA, USA; 3 Department of Family and Community Medicine, University of California, San Francisco, San Francisco, CA, USA; 4 Gladstone Institute, University of California, San Francisco, San Francisco, CA, USA; 5 San Francisco AIDS Foundation, San Francisco, CA, USA

**Keywords:** transgender women, PrEP, HIV prevention

## Abstract

**Introduction:**

Globally, transgender (“trans”) women are one of the key populations most disproportionately impacted by HIV. Pre-exposure prophylaxis (PrEP) is the newest and most promising biomedical HIV prevention intervention to date. This paper reviews relevant literature to describe the current state of the science and describes the potential role of PrEP among trans women, including a discussion of unique considerations for maximizing the impact of PrEP for this vulnerable population.

**Methods:**

Available information, including but not limited to existing scientific literature, about trans women and PrEP was reviewed and critiqued based on author expertise, including PrEP clinical trials and rollout.

**Results:**

To date, PrEP demonstration projects and clinical trials have largely excluded trans women, or have not included them in a meaningful way. Data collection strategies that fail to identify trans women in clinical trials and research further limit the ability to draw conclusions about trans women's unique needs and devise strategies to meet them. Gender-affirming providers and clinic environments are essential components of any sexual health programme that aims to serve trans women, as they will largely avoid settings that may result in stigmatizing encounters and threats to their identities. While there is currently no evidence to suggest drug-drug interactions between PrEP and commonly used feminizing hormone regimens, community concerns about potential interactions may limit interest in and uptake of PrEP among trans women.

**Conclusions:**

In scaling up PrEP for trans women, it is essential to engage trans communities, utilize trans-inclusive research and marketing strategies and identify and/or train healthcare providers to provide gender-affirming healthcare to trans women, including transition-related care such as hormone provision. PrEP implementation guidelines must consider and address trans women's unique barriers and facilitators to uptake and adherence.

## Introduction

Transgender (“trans”) women are disproportionately impacted by HIV worldwide [1]. A meta-analysis found that trans women are 49 times more likely to be living with HIV than the general population, with an estimated global pooled prevalence of 19% [2]. This overwhelming disparity calls attention to the urgent need for effective HIV prevention strategies that meet the unique needs of this population. The World Health Organization's Consolidated Guidelines for Key Populations state “the high vulnerability and specific health needs of trans people necessitate a distinct and independent status in the global HIV response” [1]. This paper reviews relevant literature to describe the current state of the science and the potential role of pre-exposure prophylaxis (PrEP) among trans women, including a discussion of unique considerations for maximizing the impact of PrEP for this vulnerable population.

Transgender identities are diverse and nuanced, varying between and within cultures. The terms “transgender” and “trans” are often used as umbrella terms to describe people whose gender identity differs from the sex assigned at birth; however, many people included in discussions of trans people may use other or additional terms to describe themselves [3] and/or may identify outside of the typical male/female binary altogether. While the terms “transgender” and “trans” are more common in Western cultures, they are also gaining more widespread use as the movement for transgender human rights becomes globalized. Some cultures acknowledge genders outside of the binary, sometimes referred to as “third gender” classifications, such as Native American *berdaches* or “Two Spirit” people, or the Fa'afafine of Samoa. Other terminology used to describe trans people includes the terms *mak nyah* in Malaysia [4], *kathoey* in Thailand [5], *hijra* in India, Bangladesh and Pakistan [6–8], *waria* in Indonesia [9], *rae rae* and *mahu* in French Polynesia [10] and *travesti* in South America [11].

Furthermore, there is great variation in access to healthcare, human rights and availability of transition-related medical care. Thus no singular, monolithic transgender identity or classification exists. For the purposes of this discussion, the terms “transgender” or “trans” women describe people who share a common experience of being assigned male sex at birth, but who identify as female, transgender or trans female, or another identity along the trans-feminine spectrum, while acknowledging that cultural context introduces variability along many dimensions of life experience. At this time, there is limited information about the feasibility, acceptability and effectiveness of PrEP for trans women cross-culturally. Much of the existing information comes from one international clinical trial where enrolment of trans women was limited to a few sites and from regional studies in North and South America and Thailand.

Despite contextual differences, trans-related stigma (“transphobia”) is pervasive cross-culturally and can limit opportunities and access to resources in a number of critical life domains (e.g. employment, healthcare), persistently affecting the physical and mental health of trans people, including HIV [12]. Trans women face unique challenges related to sex work and need for gender affirmation that can increase their vulnerability to HIV [13–15]. Worldwide, trans women who engage in sex work experience unique structural, interpersonal and individual vulnerabilities that contribute to a disproportionate risk for HIV compared with non-trans (or “cisgender,” a term often used to describe people who do not identify as transgender) male and female sex workers [16,17]. In addition, there is a clear need for increased HIV testing among trans women [18], with some preliminary evidence for feasibility and acceptability of self-testing [19]. While rates of HIV among trans men are lower than those of trans women, there is evidence of HIV risk behaviours among trans men who have sex with men (MSM) and subsequent speculation that HIV rates may increase among this population in the years to come [20,21].

Recently, PrEP has garnered a great deal of interest and attention as the newest and most promising biomedical HIV prevention intervention developed and tested to date. The first clinical trial of PrEP (iPrEx) included both high-risk MSM and trans women; risk of HIV acquisition was reduced by 44% [22]. However, a sub-analysis of the iPrEx data found no effectiveness (based on intention to treat rather than whether PrEP was actually used) among the sub-group of trans women in the study [23]. As with MSM, all of the trans women who became infected had low or undetectable drug concentrations, suggesting use of fewer than four tablets per week. The detection of PrEP drug concentrations among trans women using feminizing hormones was similar to trans women not using hormones and MSM for the first 12 weeks of the study, then dropped off afterward. This pattern suggests that long-term adherence was the issue rather than a drug-drug interaction. Lower levels of uptake and adherence among trans women compared to MSM likely contributed heavily to the differential rates of efficacy, but there have not yet been pharmacokinetic studies to rule out a drug-drug interaction of PrEP with hormones [24]. To date, iPrEx is the only clinical trial of PrEP for HIV prevention with confirmed enrolment of trans women [25]. Trans women are eligible for enrolment in the majority of PrEP studies that enrol MSM [26], including Ipergay [27] and HPTN 067 ADAPT (R. Grant, International AIDS Conference 2015, Vancouver). However, very few if any trans women have actually been enrolled in these trials [28].

PrEP demonstration projects to date have reported low or unclear levels of enrolment of trans women [28,29]. The Demo Project, a three-city PrEP demonstration project conducted in the United States, is usually described as a cohort of MSM and trans women [28]. However, the project enrolled only seven trans women across all three sites, out of a total of 557 enrolled participants. Due to low enrolment numbers, it is impossible to make comparisons or draw conclusions about unique correlates of uptake and adherence among trans women. Furthermore, there are currently no guidelines for PrEP demonstration projects that provide specific considerations for provision of PrEP to trans women. The World Health Organization guidance mentions trans women but there is no consideration of specific needs among this population. Guidance on PrEP from the US Centers for Disease Control and Prevention makes no mention of trans women whatsoever [30,31]. In 2016, the California HIV/AIDS Research Program funded the first large-scale PrEP demonstration projects specifically for trans populations, including trans men [32]. As this funding is recent, there are not yet any data available.

## PrEP awareness and acceptability among trans women

Low levels of PrEP awareness among trans women have been noted in San Francisco [33], Brazil [15], Chicago and Boston [34]. Some studies, however, have found that while PrEP awareness is low among trans women, interest in PrEP is high once patients are informed [34,35]. A study of 107 trans women and 131 MSM in Chang Mai, Thailand, found similar levels of PrEP acceptability between the two groups. However, significant differences between MSM and trans women were found in sexual behaviours, patterns of medication use and correlates of PrEP acceptability. For example, trans women were more likely to exclusively engage in receptive anal sex, which may impact their HIV risk perception and thus their willingness to take PrEP [36].

PrEP may be an empowering tool for trans women who are engaged in sex work to increase their sense of personal control over HIV prevention. Sex work is more prevalent in the lives of trans women due to social and economic marginalization, and PrEP programmes seeking to serve trans women may benefit from incorporating messaging about HIV prevention during sex work [35]. Trans women may have significant mistrust of the medical community's awareness of transgender-specific health concerns [37]. These concerns are well founded given that half of a large sample of trans people in the United States reported having to teach their provider about their own care [38]. Concerns about potential negative effects of PrEP on gender-affirming hormone therapy may also represent a barrier to PrEP acceptability [35]. An interim analysis of 608 HIV-positive trans women of colour in care at several clinics in the United States found that the perception of a negative effect of antiretroviral drugs on gender-affirming hormone therapy was associated with an odds ratio of 2.88 for having taken a higher dose of hormones than prescribed in the prior six months [39]. This finding could suggest that there is a concern regarding a negative action of ART medications on hormones, which may for some result in avoidance of or non-adherence to PrEP.

## Building trust in PrEP: gender-affirming clinical practices

Gender-affirming healthcare includes using patients’ preferred names and pronouns, respecting diversity in patients’ gender identities and expressions, and generally creating safe spaces for trans patients to be themselves, in addition to the provision of hormone and other gender-affirming medical care. Providers and clinical staff should be adequately trained to provide a safe, welcoming and culturally appropriate environment for trans people to seek care. This includes the provision of safe restrooms, comfort with and use of appropriate trans-related terminology, and the display of trans-affirming visuals and health-related information in waiting rooms, as appropriate.

Gender-affirming hormone therapy has been found to improve quality of life and social functioning and to reduce levels of anxiety and depression [40–42]. Bundling of a desired service such as gender-affirming medical interventions with HIV prevention efforts likely has synergistic value [43]. Engaging trans women in both behavioural and biomedical HIV prevention activities at the time and location of provision of gender-affirming hormone therapy fits well into the bundling model. Access to gender-affirming hormones also represents a potential intervention within several constructs of the Model of Gender Affirmation, including reducing barriers to healthcare, reduced body shame, increased access to gender affirmation and reduced use of street hormones or silicone [14].

It is essential to train healthcare providers to provide gender-affirming healthcare to trans women, including hormone provision. It cannot be assumed automatically that providers and clinic staff who serve MSM are equipped to recruit, retain and provide care to trans women [37]. Programming and services that are designed for MSM or offered through clinics that primarily serve MSM often do not meet the needs of trans women, as many trans women do not feel comfortable accessing programmes and services designed for men and these services do not address their unique life context [44]. In San Francisco, trans women described the importance of finding trans-competent, gender-affirming providers as a powerful facilitator to increasing the acceptability of PrEP [35].

While it is important to continue to develop trans-specific services, existing programmes should also be expanded to include effective programming for trans women. In low- and middle-income countries that do not have the resources to justify funding and developing separate trans-specific programming, health ministries should possess basic knowledge on effectively serving local communities of trans women at risk for acquiring HIV. For example, offering PrEP and other sexual health services to trans women through services oriented to cisgender women represents another potential approach. Many women-focused sexual health services are seeking to be more trans-inclusive, but there are currently no data or guidance available to support these efforts. Contextually situated psychosocial drivers of HIV risk among many trans women are more similar to those of non-trans women than those of men. These psychosocial drivers include experiences of trauma, domestic and sexual violence, misogyny, survival sex work, sexual objectification and unequal power in relationships to negotiate safer sex [37,38,45,46].

Where transition-related services such as hormones are not available, clinicians should still provide care that is gender affirming and assist the person in accessing transition-related care services when possible. Assistance in obtaining legal identification, health insurance and identifying trans-specific support services can all be helpful. If the patient is taking hormones obtained in other ways (i.e. on the street or Internet), clinicians can support a person's transition by checking hormone blood levels and providing education about safe hormone use.

PrEP services are amenable to algorithmic care protocols, making such services implementable in a large variety of clinical settings including sexual health clinics, family practice clinics, reproductive health clinics, community clinics and student clinics, in both small and large practices. However, clinical familiarity with PrEP protocols and sexual health testing and management is currently limited. A greater limitation arises from the lack of familiarity with best practices for gender-affirming medical care, such that finding a healthcare provider who is able to provide both PrEP and gender-affirming care has been difficult for trans people. As such, another approach to improving PrEP uptake and adherence in trans women is by expanding PrEP-related knowledge and skill among existing providers of gender-affirming clinical services.

Segmentation of sexual health services according to the gender binary is another barrier. Sexual health services oriented exclusively for MSM frequently do not provide services required by trans men, including cervical cancer screening, pregnancy tests and skill in prescribing hormonal contraception. Similarly, practices that are oriented mainly to the needs of cisgender women, such as OB/GYN practices, may not be familiar with evaluation and management of trans women or men. Expanding gender-specific services to include trans people is crucial to improving sexual health within these communities.

Further, trans women may experience unique barriers to obtaining healthcare. In addition to increased rates of unemployment, which can result in un- or under-insurance, trans women may avoid enrolling in safety net insurance programmes or visiting clinics due to lack of legal identification documents that reflect their affirmed gender. Mismatches between the sex listed on legal documents and insurance policies may result in a denial of insurance claims. While insurance coverage should not determine access to PrEP, drug costs can be a major barrier to PrEP rollout, especially in low- and middle-income countries [47].

## Building evidence for PrEP: trans-inclusive research strategies

Uniform and universal collection of gender identity data is an essential structural-level HIV prevention intervention in trans populations. Failure to identify, describe and quantify trans populations results in an invisibility at the institutional level, with resultant exclusion from policymaking, funds allocation and research activities [44]. Trans women, their advocates and public health researchers have issued a strong call for the disaggregation of trans women from MSM in HIV prevention programming and research [2,17,48], as the importance of incorporating gender-affirming practices in addressing HIV among trans women is becoming increasingly recognized [14,37].

Ongoing challenges in collecting valid data on trans people and HIV include cultural variation in language used to describe trans people and lack of standardized measurements [49]. The use of the two-step method for the collection of gender identity data has been recommended by a wide range of experts and institutions [50,51]. This method includes querying both gender identity (using a wide range of locally appropriate options) and birth-assigned sex. Trans people are identified as those with a gender identity that differs from their birth-assigned sex. Failure to use the two-step method can result in some trans people responding to a single question on sex/gender as either “male” or “female” rather than “transgender”; a study comparing these two methods found the two-step method doubled the number of trans people identified within a population [52]. The consequences of a failure to accurately record gender identity were described in the aforementioned sub-analysis of trans participants in the iPrEx trial, in which a more in-depth analysis using surrogate markers revealed that 13% of trans participants had not been identified in the original analysis [24,53].

Low enrolment of trans participants in clinical trials of PrEP to date can likely be attributed to the passive inclusion of trans women without implementation of any trans-specific recruitment strategies or proactively training staff to provide care that is sensitive and supportive to trans people. Enrolment of trans women in future trials can be improved by carefully planning study outreach efforts and intentionally designing recruitment and retention strategies to address the unique needs and potential concerns of trans women. Designing gender-affirming strategies, such as specifically addressing and representing trans women in recruitment materials, hiring trans study staff, offering gender-affirming healthcare and ensuring the representation of trans people at all levels of the project, such as on advisory boards, study staff and involving trans people in the design of the study, can all help improve enrolment of trans people in clinical trials and future PrEP demonstration projects [54].

## Building demand for PrEP: community engagement and empowerment

While some initial research has shown that there is interest in PrEP among trans women when they are aware of it, demand for PrEP among trans women is yet unproven. Building demand for PrEP will be an important first step in implementation, and community engagement in this process will be crucial. Community mobilization strategies are particularly effective in increasing empowerment and decreasing stigma among marginalized populations and in disseminating novel information via trusted social networks [55]. Social marketing strategies should emphasize gender-affirming, sex-positive messaging about the potential benefits of PrEP, such as increased sexual pleasure and intimacy, increased sense of safety during sex, decreased HIV-related anxiety, decreasing stigmatizing attitudes toward HIV-positive partners, increased sense of community and increased self-efficacy for HIV prevention [56]. PrEP can empower the individual to take control of preventing their own acquisition of HIV without relying on prevention strategies often controlled by one's partner (i.e. condom use), and the disclosure of one's own decision to take PrEP is also a personal choice. PrEP champions, or individuals who are willing to publicly share about their own positive experiences with PrEP, may be particularly powerful sources of support and builders of trust when they come from within local trans communities.

Involvement of trans women in the design and development of PrEP studies and programmes that aim to include them is crucial to successful enrolment. This helps to ensure that the messaging, environment and questions being asked are relevant to trans women's unique needs and concerns. Patient decision-making tools and adherence support strategies should be designed specifically for trans women with their input, rather than adapted from strategies that were designed for MSM.

## Biomedical considerations

No evidence currently exists to suggest that PrEP interacts with commonly used feminizing hormone regimens, and evidence from studies of antiretroviral interactions with hormonal contraceptives has been reassuring [57]. However, there have not yet been any pharmacokinetic studies of these potential drug-drug interactions with trans women. Studies of interactions between combined oral contraceptives and antiretroviral medications have generally not indicated negative interactions between the classes [58]. Important differences exist in the context of transgender care, where a range of oestrogens are used, most commonly bioidentical 17-beta estradiol, in contrast to the synthetic ethinyl estradiol used in oral contraceptives. Furthermore, use of progestogens is inconsistent among trans women. Further study is needed to explore potential interactions between PrEP medications and commonly used gender-affirming hormones. Also requiring exploration are any changes in the anal epithelium in response to hormone therapy, as well as considerations in trans women who have undergone a penile inversion vaginoplasty, in which penile and scrotal skin is inverted to create a vagina and vulva, possibly with the use of urethral mucosa. The risks of HIV transmission via these organs, as well as the risks and rates of ulcerative genital infections and local concentrations of PrEP medication are unknown and require further study [17].

As previously described, compared with MSM, trans women in the iPrEx study had lower concentrations of active metabolites of tenofovir disoproxil fumarate (TDF) and emtricitabine [53]. Concentrations of PrEP medications were especially low among trans women reporting use of feminizing hormones, which may reflect less PrEP use or a drug-drug interaction. While there are no systemic drug-drug interactions between TDF and oral contraception, there are known interactions at the level of drug transporters between these classes of medications that could affect drug concentrations in target tissues [59]. Drug-drug interactions between TDF and either natural oestrogens or anti-androgenic agents used for gender-affirming hormone therapy among trans women have not been studied. Drug-drug interactions between emtricitabine and any of these medications have also not been studied.

## Conclusions

The future of PrEP among trans women relies on reversal of the relative invisibility of trans women in research and clinical services that inform the structure of programming and access to PrEP. Guidelines developed for the implementation of PrEP must consider the unique barriers to and facilitators of uptake among trans women. Risk assessment tools, adherence support and retention strategies are currently being developed without consideration of issues unique to trans women and are not validated for use with trans women [60]. Structural interventions are needed, such as comprehensive provider training programmes for all level staff to better serve the needs of trans women and increase service utilization and improve wellbeing, while effecting lasting institutional change [61]. It is vitally important that PrEP messaging and information be delivered via trans-specific networks with the unique concerns and life contexts of trans women in mind. Community-based strategies, such as community mobilization to increase knowledge and trust of information about PrEP among trans women, should be explored.

Because trans women may prioritize hormone therapy over other healthcare [35,37], bundling of PrEP with gender-affirming hormones may help address this barrier to PrEP uptake and adherence by both serving as a venue for distribution of accurate information regarding drug interactions and co-packaging of hormones and PrEP medication to overcome any barriers relating to increased pill burden. Multimodal interventions are recommended to increase PrEP uptake and provide adherence support, and all interventions must consider culturally unique barriers to healthcare access and adherence to maximize effectiveness with trans women [30,60,62]. PrEP can be a cost-effective addition to comprehensive sexual health programmes when people with the highest risk of acquiring HIV, such as trans women, are given priority of access [47]. PrEP may also provide additional benefits above and beyond HIV prevention, such as community empowerment and engaging previously marginalized communities of trans women into healthcare. The meaningful engagement and inclusion of trans women in the development of PrEP rollout strategies and the disaggregation of trans women from MSM in ongoing PrEP research and programming are essential initiatives to promote this highly promising HIV prevention tool among a key population that to date has been largely overlooked.
